# GPR56 facilitates hepatocellular carcinoma metastasis by promoting the TGF-β signaling pathway

**DOI:** 10.1038/s41419-024-07095-6

**Published:** 2024-10-01

**Authors:** Yiming Luo, Junli Lu, Zhen Lei, Dean Rao, Tiantian Wang, Chenan Fu, He Zhu, Zhiwei Zhang, Zhibin Liao, Huifang Liang, Wenjie Huang

**Affiliations:** 1 Hepatic Surgery Centre, Tongji Hospital, Tongji Medical College, Huazhong University of Science and Technology, Hubei Key Laboratory of Hepato-Pancreato-Biliary Diseases, Wuhan, Hubei 430030 China; 2 Clinical Medicine Research Center for Hepatic Surgery of Hubei Province; Key Laboratory of Organ Transplantation, Ministry of Education and Ministry of Public Health, Wuhan, Hubei 430030 China

**Keywords:** Liver cancer, Targeted therapies, Metastasis

## Abstract

The metastasis of hepatocellular carcinoma (HCC) poses a significant threat to the survival of patients. G protein-coupled receptor 56 (GPR56) has garnered extensive attention within malignant tumor research and plays a crucial role in cellular surface signal transmission. Nonetheless, its precise function in HCC remains ambiguous. Our investigation reveals a notable rise in GPR56 expression levels in human HCC cases, with heightened GPR56 levels correlating with unfavorable prognoses. GPR56 regulates TGF-β pathway by interacting with TGFBR1, thereby promoting HCC metastasis. At the same time, GPR56 is subject to regulation by the canonical cascade of TGF-β signaling, thereby establishing a positive feedback loop. Furthermore, the combination application of TGFBR1 inhibitor galunisertib (GAL) and GPR56 inhibitor Dihydromunduletone (DHM), significantly inhibits HCC metastasis. Interventions towards this signaling pathway could offer a promising therapeutic approach to effectively impede the metastasis of GPR56-mediated HCC.

## Introduction

HCC is a prevalent malignancy, ranking third in global cancer-related fatalities [1]. Elevated mortality rates arise due to delayed detection in advanced disease stages, frequent metastasis of tumors, and recurrence of tumors following surgical removal [2]. Even with notable progress in recent decades, patient outcomes in late-stage HCC remain suboptimal. Hence, comprehending the intricate molecular mechanisms governing HCC development and metastasis is paramount.

The TGF-β pathway plays multiple roles in liver disease and hepatocellular carcinoma (HCC), both promoting liver fibrosis and inflammation and inhibiting tumor proliferation and promoting metastasis [3]. The TGF-β protein binds to the receptor kinase and initiates a phosphorylation-dependent signaling cascade that mainly includes downstream signaling of the SMAD protein [4, 5]. In the advanced stages of cancer progression, TGF-β can promote tumor metastasis by stimulating tumor cells to undergo epithelial-mesenchymal transformation (EMT), which plays a pro-cancer role [6, 7]. Our previous research delved into the TGF-β1/SMAD3 classical signaling mechanism, elucidating the role of the downstream molecules PTPRε and JunBP in facilitating HCC migration and metastasis [8, 9].

G protein-coupled receptors (GPCRs) represent one of the largest membrane protein families, typically composed of large extracellular domains (ECDs), seven-transmembrane domains (7TMs), and intracellular domains (ICDs) [10]. Abnormally activated GPCRs are commonly linked to various aspects of cancer, encompassing tumor proliferation, viability, angiogenesis, and metastasis [11, 12]. As over 30% of drugs currently target GPCRs, understanding their biological functions in malignant tumors is paramount [13]. Among GPCRs, GPR56 has been extensively studied in a variety of diseases [14, 15]. Zendman et al. first reported downregulation of GPR56 in highly metastatic melanoma cell lines compared to low metastatic counterparts in 1999 [16]. Subsequent research has delved into various aspects of GPR56’s role in malignancies, spanning from its differential expression to regulatory mechanisms. Numerous studies indicate elevated expression of GPR56 in various tumor tissues, including esophageal squamous cell carcinoma, glioblastoma, and human fibrosarcoma [17–19]. GPR56 plays a crucial role in a range of biological processes, including tumor growth, cell migration, angiogenesis, adhesion, programmed cell death and cell cycle progression [17, 20, 21]. Due to its significant N-terminal fragment (NTF), GPR56 plays pivotal roles in various malignancies through its interactions with vascular endothelial growth factor (VEGF), collagen III, CD81, and transglutaminase 2 (Tg2) [22–26].

This study reveals the mechanism by which GPR56 enhances TGF-β signaling to facilitate HCC metastasis through its interaction with TGFBR1. Furthermore, we provide evidence that TGF-β1/SMAD3 transactivates the expression of GPR56 directly. Importantly, our findings highlight the effectiveness of combining the GPR56 inhibitor DHM with the TGFBR1 inhibitor GAL to mitigate HCC metastasis.

## Materials and methods

### Clinical samples

Approval for this study was granted by the Ethics Committee of Tongji Medical College. Tumor and paired tissues adjacent to the carcinoma were gathered from 40 patients diagnosed with HCC who underwent clinical surgery at the Hepatic Surgery Center, Tongji Hospital (Wuhan, China) between 2014 and 2015. Furthermore, additional 123 cases of clinical and pathological follow-up data for patients with HCC (between 2012 and 2015) were provided by the Hepatic Surgery Center, Tongji Hospital (Wuhan, China). All patients, who provided informed consent, were followed for up to 65 months.

### Cell lines and culture

HLF and HCC-LM3 were obtained from the China Center for Type Culture Collection (CCTCC). PLC/PRF/5 (ALEX) was obtained from Procell Life Science & Technology Co., Ltd., with the catalog number XB0422-0388. The human normal liver cell line, the hepatoblastoma cell lines HepG2, HCC cell lines Huh7, Hep3B, MHCC-97H, and HEK293T (293 T) were obtained from the Hepatic Surgery Center, Tongji Hospital, Huazhong University of Science and Technology (HUST). The cell lines underwent cultivation in Dulbecco’s Modified Eagle Medium (DMEM), with an additional supplementation of 10% fetal bovine serum (FBS) procured from Pricella Life Science & Technology Co., Ltd. The cells were incubated at 37 °C in a humidified atmosphere with 5% CO2. The types of small molecule compounds used in cells and the concentrations used can be found in supplementary Table S2.

### Western blotting (WB)

After washing with PBS buffer, cells were subjected to lysis using RIPA buffer containing both protease inhibitor cocktail and phosphatase inhibitor cocktail (MedChemExpress, USA). This lysis process was conducted at a temperature of 4 °C for a duration of 30 min. The electrophoresis solution is configured with tris-HCI, glycine and SDS (Beijing Dingguo changsheng Biotechnology Co.,Ltd). Equal quantities of proteins underwent separation via SDS-PAGE, followed by their transfer onto polyvinylidene fluoride (PVDF) membranes (Millipore, USA). Following this, the membranes underwent blocking with 5% bovine serum albumin (BSA) at 37 °C for a duration of 1 h. Subsequently, the membranes were subjected to overnight incubation at 4 °C with a specific primary antibody, followed by incubation the next day with a secondary antibody for 1 h at 37 °C. Visualization of the membranes was achieved using an ECL detection system from Bio-Rad Laboratories. Information on all antibodies used is in the Supplementary Table S3.

### Immunohistochemistry (IHC)

After fixation with 4% formaldehyde at room temperature for at least 1 day, the tumour tissue was embedded and sectioned using paraffin. The paraffin sections were initially heated to 65 °C and baked for a duration of 30 min. Subsequently, they underwent deparaffinization in ethanol, employing a decreasing concentration gradient. Antigen restoration was conducted using either 0.01 M sodium citrate buffer (with a pH of 6.0) or an EDTA solution (with a pH of 9.0). Endogenous peroxidase activity was neutralized by washing thrice with PBS and subsequently immersing in 3% H2O2 for a duration of 15 min. Subsequent to this, blocking was achieved by treating with 5% bovine serum albumin for 60 min at 37 °C, followed by an overnight incubation in a chamber with appropriate humidity containing the primary antibody. Following this, the tissue sections underwent incubation with a secondary antibody conjugated with horseradish peroxidase (HRP) for 45 min at ambient temperature. Antibody binding was visualized using DAB, and the reaction was stopped upon the appearance of a brown color by immersing the tissue sections in distilled water.

Scoring for immunohistochemical staining and in situ hybridization was conducted by assessing staining intensity alongside the proportion of tumor cells exhibiting positive staining. Staining intensity was assessed on a scale ranging from 0 to 3, with 0 representing colorless, 1 indicating yellowish, 2 representing brown, and 3 indicating tan. Positively stained cells were scored as follows: 0 for less than 5% of total cells stained, 1 for 10%-25%, 2 for 26%-50%, 3 for 51%-75%, and 4 for more than 75% of positively stained tumor cells. The final staining score was derived by multiplying the staining intensity score by the percentage of positively stained cells.

### Cell infection

HCC cells were spread in six-well plates at 50% density and incubated overnight at 37 degrees to make them adherent. Lentivirus was constructed by pLV, psPAX2, pMD2.G and other plasmids. Plasmids was extracted by using A Steadypure Plasmid DNA Extraction Kit (Accurate Biotechnology (Hunan) Co.,Ltd, ChangSha, China) was used to extract Plasmids. Transfection with plasmid was performed using FectinMore™ (Chamot Biotechnology LTD). The infected cells were screened in a medium containing 5 μg/ml purinomycin for a duration of 2 weeks. Sequences of small hairpin RNAs (shRNAs) targeted for gene knockdown in Supplementary Table S4.

### Animals

Male BALB/c nude mice, aged 4 weeks, were procured from Hubei Biont Biological Technology Co., Ltd, and raised under pathogen-free environments. Individuals with similar body weight were selected and randomly grouped into 5 individuals per cage. In the in vivo metastasis assay, 10^6^ cells were added to 100 μl of serum-free DMEM and injected into the tail vein of nude mice. After a span of 2 months, the nude mice were executed and the lungs were dissected. All metastases in the lungs were counted to assess the development of lung metastases. In the intrahepatic tumor implantation model, 1 × 10^6^ cells were resuspended in 30 μL of serum-free DMEM and subsequently implanted beneath the capsule of the right liver lobe. Tumors were excised after 4 weeks.

### RNA extraction and Real-time PCR

Total RNA was extracted from patient tissues and cell lines utilizing the FastPure® Cell/Tissue Total RNA Isolation Kit V2 (Vazyme Biotech Co.,Ltd). Subsequently, 2 µg of isolated RNA was transcribed to cDNA using the HiScript III Q Select RT SuperMix for qPCR (Vazyme Biotech Co.,Ltd). Quantitative real-time PCR was performed using 2 xQ3 SYBR qPCR Master Mix (Universal) (#22204, Tolo Biotech Co.,Ltd). Primer sequences used for real-time qPCR were listed in the Supplementary Table S4.

### Wound healing assay

HCC cells were plated in 6-well dishes when reaching 90 to 100% confluence. Confluent monolayers of HCC cells were cultured overnight and scratched using a 10 µl pipette tip to simulate an artificial wound. Subsequently, the cells underwent three rinses with PBS to eliminate any dislodged cells and cellular debris. At 0 and 48 h after scratching, the distance of cell migration was recorded using a phase contrast microscopy. Quantitative analysis involved capturing images of five randomly selected regions. Each experimental trial was replicated a minimum of three times.

### Transwell assays

The upper chamber membrane was initially coated with a 1:4 blend of matrigel and DMEM, incubated at room temperature for 4 h. Next, the upper chamber received a seeding of 3 × 10^4^ cells resuspended in 200 µl of serum-free DMEM, while 600 µl of DMEM containing 10% FBS was added to the lower chamber. Following incubation for 24 to 48 h at 37 °C with 5% CO2, non-migratory or non-infected cells residing on the filter membrane were gently removed from the upper surface. The migrated cells were fixed under 4% formaldehyde for 30 min and then stained with 0.1% crystal violet for 15 min. Cell enumeration was performed under a light microscope. Each experiment was conducted with a minimum of three replicates.

### Co‑immunoprecipitation (co‑IP)

Cell lysis was performed for 30 min at 4 °C using IP-lysis buffer. This buffer also included both protease inhibitor and phosphatase inhibitor cocktails sourced from MedChemExpress, USA. Subsequently, the lysed cells underwent centrifugation at 12 000 rpm for 15 min at 4 °C. The resultant supernatant, containing the proteins, was subjected to immunoprecipitation using the specified antibodies and allowed to incubate at 4 °C. The next day, the lysate was mixed with Magnetic Agarose Beads (Biolinkedin, Shanghai, China) and incubated for 2–4 h, followed by three washes with IP wash buffer. The Magnetic Agarose Beads were then combined with 2× sampling buffer and heated at 95 °C for 10 min before being subjected to analysis via Western blotting.

### Immunofluorescence (IF) analysis

The cells were initially exposed to 4% paraformaldehyde for 15 min to induce fixation. Subsequently, they underwent three consecutive washes with PBS and were then subjected to permeabilization using a 0.5% Triton X-100 solution for a duration of 10 min. Following this, the cells were blocked with 3% BSA for 60 min at 37 °C, followed by overnight incubation at 4 °C with primary antibodies. The next day, after washing the cells three times with PBS, the cells were incubated with fluorescent secondary antibody for 1 h at room temperature and protected from light. Finally, the nuclei were stained with DAPI (Wuhan Goodbio Biotechnology Co., Ltd., Wuhan, China) for 8 min.

### Dual‑Luciferase reporter assay

Luciferase function was assessed through the application of the dual luciferase reporter assay (Promega, Madison, WI, USA). Utilizing a GloMax 20/20 Luminometer (Promega), the relative luciferase activity was quantified. Subsequently, luciferase activity was adjusted based on Renilla activity levels.

### RNA-sequencing (RNA-seq)

RNA-sequencing and data analysis services were provided by Wuhan Generead Biotechnologies Co. Ltd. To identify genes that were differentially expressed, we set a cutoff value of log2 (fold change) >1 and p < 0.05.

### Statistical analysis

Mean values were depicted with their respective standard deviations. Each experimental trial was conducted independently, repeating three times or more for biological reproducibility. To assess the significance between two groups, Student’s t-tests (for normally distributed data) or Wilcoxon signed-rank tests (for paired comparisons) were utilized. In cases involving multiple groups, statistical scrutiny employed either one-way ANOVA or two-way ANOVA. Immunohistochemical scoring underwent analysis via the chi-squared test. Survival curves were visualized using the Kaplan-Meier analysis, and their significance was gauged through the log-rank test. Pearson correlation tests were employed to assess correlations. All statistical analyses were performed with GraphPad Prism 8.0 software, considering p < 0.05 as statistically significant (*p < 0.05, **p < 0.01), while “ns” denotes no significance.

## Results

### Increased GPR56 expression demonstrated a correlation with clinical outcomes among HCC patients

**Fig. 1:**
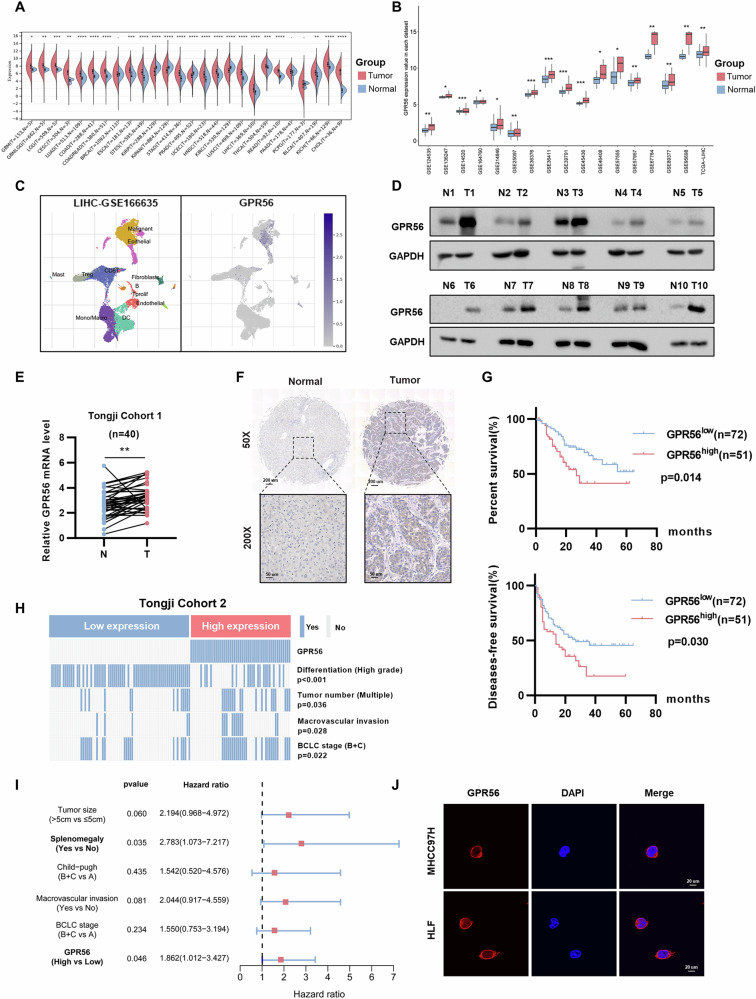
Increased GPR56 expression demonstrated a correlation with clinical outcomes among HCC patients. **A** The GPR56 expression in pan-cancer. **B** The expression of GPR56 in 17 datasets of HCC from TCGA and GEO. **C** Expression of GPR56 in single cell sequencing dataset GSE166635. **D**, **E** Protein and mRNA levels of GPR56 in 40 paired HCC samples detected by WB and real-time qPCR. **F** Representative images of GPR56 IHC staining in normal tissues compared to HCC tissues. Scale bar: 200 µm (up), 50 µm (bottom) (**G**) Kaplan-Meier diagrams illustrating overall survival (OS) rates and recurrence-free survival (RFS) in different GPR56 expression groups in Tongji cohort. **H** Chi-square analysis of the relevance of GPR56 expression with differentiation, tumor number, macrovascular invasion and BCLC stage in HCC patients. **I** Multivariate regression analyses forest plot for HCC patients from the Tongji cohort. **J** Subcellular localization of GPR56 by immunofluorescence (IF). The representation includes data and error bars, demonstrating the mean ± standard deviation from three separate independent trials. Significance levels are indicated as follows: *p < 0.05, **p < 0.01, ***p < 0.001, and “ns” indicating no statistical significance. The data were analyzed utilizing Student’s t-tests.

Firstly, we analyzed high-throughput sequencing data from 26 different tumor types available in The Cancer Genome Atlas (TCGA) database. By comparing the expression levels of GPR56 between tumor tissues and adjacent normal tissues, we found that GPR56 is upregulated in the majority of cancers (Fig. 1A). In HCC, we conducted further investigations. Subsequently, we collected 17 public datasets of HCC from TCGA and GEO, and GPR56 mRNA expression level in tumor tissues was markedly elevated compared to that in adjacent normal tissues (Fig. 1B). Furthermore, analysis of single-cell sequencing data from GSE166635 in HCC showed significant enrichment of GPR56 in malignant epithelial cell (Fig. 1C).

Validation was performed on 40 pairs of HCC tissues and matched adjacent tissues collected from the Hepatic Surgery Centre at Tongji Hospital, Wuhan. Real-time qPCR results demonstrated significant upregulation of GPR56 mRNA levels in HCC compared to adjacent tissues, which was corroborated at the protein levels through western blot analysis (Fig. 1D, E). Subsequently, immunohistochemical (IHC) staining was conducted on a patient cohort (n = 123) from Tongji hospital, revealing significantly higher expression levels of GPR56 in HCC tumor tissues compared to adjacent tissues (Fig. 1F). Further statistical analysis classified the 123 HCC samples into groups of high and low GPR56 expression, determined by staining intensity. We found that the overall survival (OS) and disease-free survival (DFS) of HCC patients in the GPR56 high-expression group were lower than patients in the low-expression group by Kaplan-Meier analysis (Fig. 1G). Additionally, GPR56 expression was closely associated with HCC differentiation degree, tumor number, macrovascular invasion, and BCLC stage (Fig. 1H and Supplementary Table S1). We employed COX regression analysis to investigate whether the expression level of GPR56, along with other risk factors, influences the survival time of HCC patients. Both univariate and multivariate COX regression analyses demonstrated that GPR56 is an independent prognostic factor for HCC, with higher expression levels being associated with poorer prognosis (Fig. 1I and Supplementary Fig. S1A). Together, these findings suggest heightened expression of GPR56 in HCC, proposing its potential utility as a prognostic indicator for individuals afflicted with this condition.

Furthermore, real-time qPCR and western bolt assays on normal hepatocyte cell lines and HCC cell lines showed that GPR56 protein and mRNA levels were higher in HCC cells than in normal human hepatocytes (Supplementary Fig. S1B–C). Subsequent subcellular localization analysis using confocal microscopy revealed predominant expression of GPR56 on the cell membrane (Fig. 1J).

### GPR56 facilitated the metastatic progression of HCC in both in vitro and in vivo

**Fig. 2:**
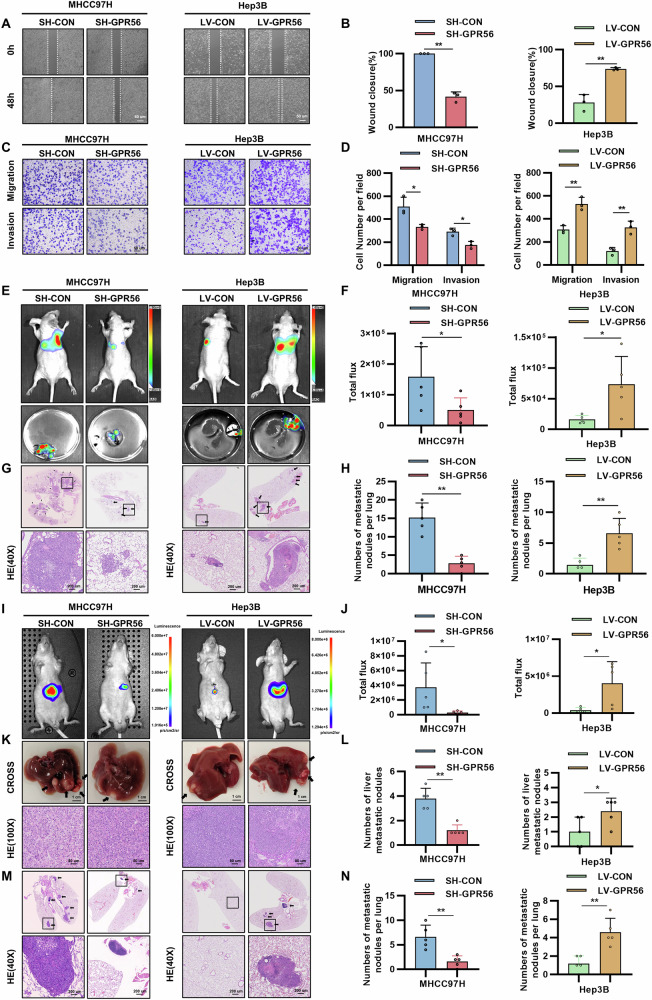
GPR56 facilitated the metastatic progression of HCC in both in vitro and in vivo. **A**, **B** Representative images (**A**) and the rate of wound closure (**B**) from the wound healing assay with MHCC97H and Hep3B. **C**, **D** Representative images (**C**) and number of cells (**D**) from migration and invasion assays with MHCC97H with MHCC97H and Hep3B. **E**–**H** Fluorescence images representative of the samples (**E**), radiance (**F**), representative HE images (**G**), and count of lung metastasis nodules (H) from SH-VEC, SH-GPR56, LV-CON and LV-GPR56 groups in tail vein injection metastasis model; scale bar: 2 mm (4×), 200 mm (40×). **I**–**N** Fluorescence images representative of the samples (**I**), radiance (**J**), gross image (**K**), count of HCC metastasis nodules (**L**), representative HE images of lung metastasis(M), number of lung metastasis nodules (**N**) from SH-VEC, SH-GPR56, LV-CON and LV-GPR56 groups in the orthotopic liver model metastasis model; scale bar: 1 cm (**K**), 200 µm (40×), 80 µm (100×). The representation includes data and error bars, demonstrating the mean ± standard deviation from three separate independent trials. Significance levels are indicated as follows: *p < 0.05, **p < 0.01, ***p < 0.001, and “ns” indicating no statistical significance. The data were analyzed utilizing Student’s t-tests.

To further investigate the biological effects of GPR56 on HCC, we stably overexpressed GPR56 in Hep3B cells with low expression levels using lentivirus and stably knocked down GPR56 in MHCC97H and HLF cells with high expression levels using three different shRNAs, selecting the shRNA-2 for further experiments. (Supplementary Fig. 2A–D). We found that GPR56 overexpression enhanced the migration and invasion capacity of Hep3B cells by wound healing and transwell assays, whereas GPR56 knockdown attenuated these capabilities in MHCC97H and HLF cells (Fig. 2A–D and Supplementary Fig. 2E, F). These results collectively confirmed that GPR56 promotes HCC migration and invasion. Furthermore, EMT markers were detected on Western blots and we found that upregulation of GPR56 in HCC cells promoted the expression of Vimentin and Occludin while inhibiting E-cadherin expression, indicating the ability of GPR56 to facilitate EMT in HCC cells (Supplementary Fig. 2G). Additionally, we employed a small molecule inhibitor, DHM, which specifically targets GPR56, inhibiting its functional activity. DHM was utilized in MHCC97H and Hep3B cell lines, resulting in a notable reduction in the migratory and invasive potential of HCC cells, as evidenced by wound healing and transwell assays (Supplementary Fig. 3A–D).

We further validated the oncogenic function of GPR56 in vivo. We established both a tail vein lung metastasis model and an orthotopic liver implantation tumor model in nude mice. The tail vein lung metastasis experiment showed that the fluorescence intensity and the number of lung implants were notably diminished in the SH-GPR56 group compared to the SH-CON group. Conversely, in the OE-GPR56 group, both fluorescence intensity and the number of lung implants exhibited elevation compared to the LV-CON group (Fig. 2E–H). Subsequently, in the SH-GPR56 group, the orthotopic liver metastasis model exhibited significantly lower fluorescence intensity, fewer tumors, prolonged survival time, and reduced lung metastasis compared to the SH-CON group. Conversely, the OE-GPR56 group showed the opposite results, with higher fluorescence intensity, more tumors, increased lung metastasis nodules, and shorter survival time compared to the LV-CON group (Fig. 2I–N). Based on these results, we conclude that GPR56 promotes the progression of liver cancer and enhances metastasis.

### GPR56 exhibited upregulation of the TGF-β pathway and interaction with TGFBR1

**Fig. 3:**
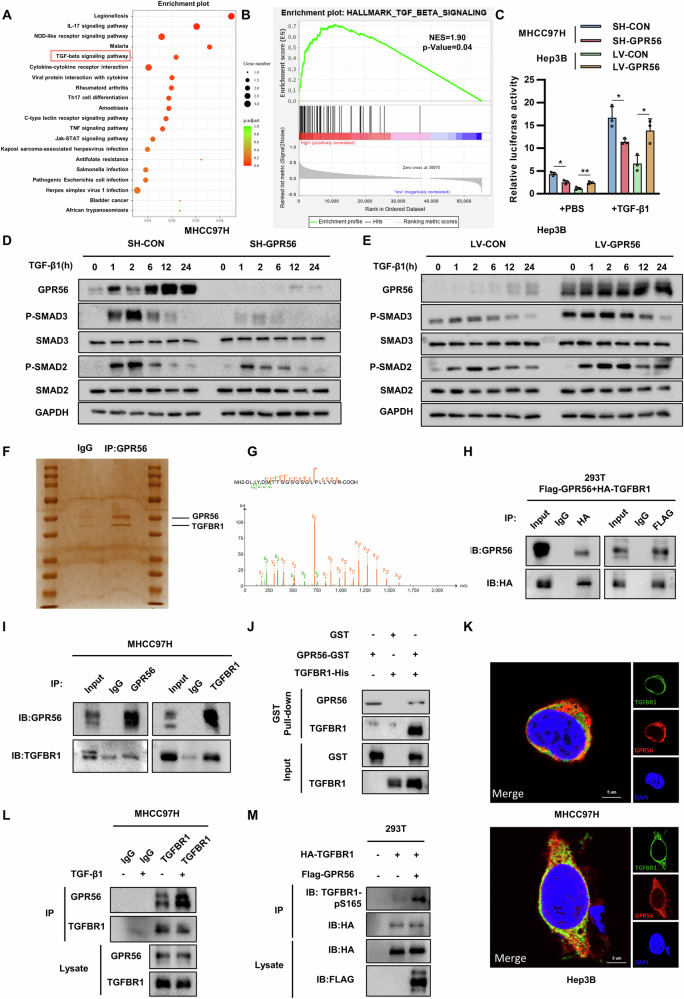
GPR56 exhibited upregulation of the TGF-β pathway and interaction with TGFBR1. **A** KEGG analysis of differentially expressed genes between GPR56 overexpressing cells and control cells. **B** GSEA analysis of TGF-β pathway grouped by GPR56 expression in TCGA-LIHC. **C** Relative luciferase activity of SBE in different groups. **D**, **E** Expression of p-SMAD2/3 in TGF-β1-stimulated GPR56 knockdown MHCC97H and GPR56 overexpressing Hep3B. **F** Silver staining of GPR56 overexpressing cells. **G** The peptide fragment of TGFBR1 identified through mass spectrometric analysis. **H** Co-immunoprecipitation (Co-IP) and western blot of exogenous proteins GPR56 and TGFBR1 in 293 T cells. **I** Co-IP and western blot of endogenously proteins GPR56 and TGFBR1 in MHCC97H cells. **J** Purified recombinant gpr56 shows direct interaction with TGFBR1 in GST pull-down assay. **K** Representative confocal immunofluorescence images (bar = 5 μm). **L** Endogenous Co-IP experiments in MHCC97H cells, with or without TGF-β1 treatment for 1 h. **M** The expression of TGFBR1-pS165 in 293 T cells. The representation includes data and error bars, demonstrating the mean ± standard deviation from three separate independent trials. Significance levels are indicated as follows: *p < 0.05, **p < 0.01, ***p < 0.001, and “ns” indicating no statistical significance. The data were analyzed utilizing Student’s t-tests.

To investigate the mechanisms of GPR56-mediated HCC metastasis, we utilized RNA-seq technology to examine mRNA changes induced by GPR56 overexpression in Hep3B liver cancer cells. Following GPR56 overexpression, pathway enrichment analysis employing the Kyoto Encyclopedia of Genes and Genomes (KEGG) unveiled significant enrichment within the TGF-β signaling pathway (Fig. 3A). Additionally, gene set enrichment analysis of TCGA-LIHC demonstrated that TGF-β signaling pathway was also significantly enriched in the GPR56 high-expression group (Fig. 3B). These results indicate that GPR56 in HCC cells may be able to activate the TGF-β signalling pathway. The physiological effects of TGF-β occur via high-affinity receptors, namely serine-threonine kinase receptors (TβR-I and TβR-II), triggering the SMAD-dependent signaling pathway. This cascade culminates in the transcriptional control of gene promoters containing the SMAD-binding element (SBE), commonly referred to as the CAGA box [27]. We assessed the effect of GPR56 on SBE luciferase activity in HCC cells, which reflects the activation of TGF-β signaling. Our findings revealed reduced SBE-luciferase activity in SH-GPR56 MHCC97H cells and enhanced activity in oe-GPR56 Hep3B cells, suggesting that GPR56 facilitates the TGF-β signaling cascade in HCC cells (Fig. 3C). In addition, immunohistochemical staining of orthotopic liver tumors in mice showed that p-SMAD3 was weakened in the SH-GPR56 group compared to the SH-CON group, while it was stronger in the LV-GPR56 group than in the LV-CON group, confirming the above results (Supplementary Fig. 4A). Furthermore, knockdown of GPR56 or its inhibition by DHM treatment mitigated the increase in phosphorylated SMAD2/3 levels triggered by TGF-β1 stimulation (Fig. 3D and Supplementary Fig. 4B). Conversely, overexpression of GPR56 promoted TGF-β1-induced Smad2/3 activation in Hep3B cells (Fig. 3E).

To further elucidate the mechanism by which GPR56 promotes the TGF-β signaling pathway, we sought to find binding partners for GPR56. Through immunoprecipitation followed by silver staining and mass spectrum, we found that TGF-β receptor 1 (TGFBR1) may be one of the potential interacting partners of GPR56 (Fig. 3F, G). TGFBR1 is a crucial component of the TGF-β signaling pathway. Co-immunoprecipitation assays and GST pull-down experiments illustrated the interaction between GPR56 and TGFBR1, indicating their binding affinity (Fig. 3H–J). We confirmed the spatial co-localization of exogenous GPR56 and TGFBR1 in wild-type 97H and Hep3B cells using immunofluorescence and laser confocal scanning (Fig. 3K and Supplementary Fig. 4C). As a membrane receptor, GPR56 possesses the capability to facilitate membrane receptor activation, degradation, or modulation of receptor kinase activity [26, 28]. We hypothesized whether GPR56 might exert regulatory influence on the activity of TGFBR1. No discernible regulation was observed in real-time qPCR and Western blot analyses between GPR56 and TGFBR1 (Supplementary S4E, F). Furthermore, TGF-β1 treatment augmented the interaction between GPR56 and TGFBR1 (Fig. 3L). Additionally, co-IP experiments showed that overexpression of GPR56 increased TGFBR1 phosphorylation (Fig. 3M). Together, these results suggest that GPR56 may mediate TGF-β1-induced TGFBR1 activation by acting as a coreceptor.

### GPR56 promoted HCC metastasis through TGFBR1

**Fig. 4:**
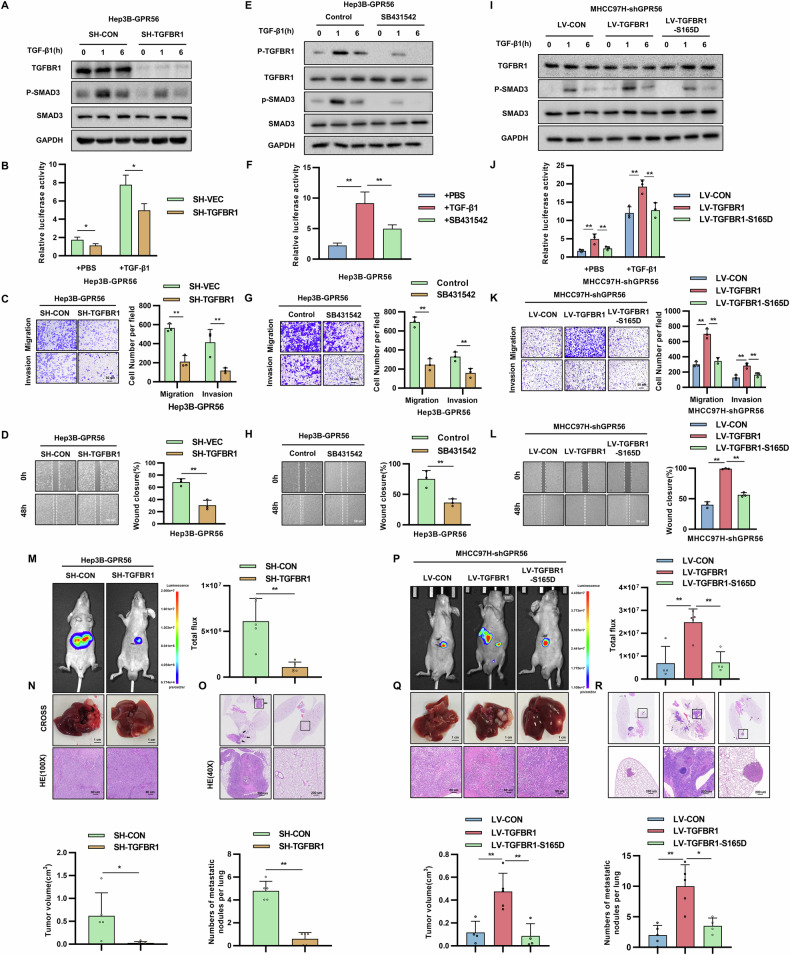
GPR56 promoted HCC metastasis by TGFBR1. Western blot (**A**), SBE luciferase activity (**B**), representative images of wound healing experiments (**C**) and representative images of migration or invasion assays (**D**) after TGFBR1 knockdown in LV-GPR56 Hep3B cells. **E**–**H** Western blot (**E**), SBE luciferase activity (**F**), representative images of wound healing experiments (**G**) and representative images of migration or invasion assays (**H**) in Hep3B cells treated with TGFBR1 phosphorylation inhibitor SB431542. **I**–**L** Western blot (**I**), SBE luciferase activity (**J**), representative images of wound healing experiments (**K**) and representative images of migration or invasion assays (**L**) after TGFBR1-WT or TGFBR1-S165D overexpression on the basis of GPR56 knockdown of MHCC97H cells. **M**–**O** Representative fluorescence images (**M**), gross image (**N**), representative HE images of lung metastasis (**O**) after TGFBR1 knockdown in LV-GPR56 Hep3B cells in the orthotopic liver metastasis model. **P**–**R** Representative fluorescence images (**P**), gross image (**Q**), representative HE images of lung metastasis (**R**) after TGFBR1-WT or TGFBR1-S165D overexpression on the basis of GPR56 knockdown in the orthotopic liver model metastasis mode comprised of MHCC97H cells. Scale bar: 1 cm (**H**, **K**), 200 µm (40×), 80 µm (100×), 50 µm (200×). The representation includes data and error bars, demonstrating the mean ± standard deviation from three separate independent trials. Significance levels are indicated as follows: *p < 0.05, **p < 0.01, ***p < 0.001, and “ns” indicating no statistical significance. The data were analyzed utilizing Student’s t-tests.

We further investigated the role of TGFBR1 in the promotion of HCC cell metastasis by GPR56. In Hep3B LV-GPR56 cells, knockdown of TGFBR1 decreased p-SMAD3 expression (Fig. 4A). Similarly, in luciferase reporter gene assays, we found that knockdown of TGFBR1 inhibited the increase in SBE luciferase activity caused by GPR56 overexpression (Fig. 4B). By wound healing assay and transwell assay, we found that knockdown of TGFBR1 eliminated the promotion of migration and invasion by GPR56 (Fig. 4C–D). Furthermore, we used the small-molecule inhibitor SB431542 to inhibit TGFBR1 phosphorylation in Hep3B cells overexpressing GPR56. We observed that when TGFBR1 phosphorylation is inhibited, the migration and invasion abilities of HCC cells are similarly suppressed (Fig. 4E–H). These findings suggest that the regulation of the TGF-β signaling pathway by GPR56 is dependent on TGFBR1. In addition, we constructed MHCC97H cells overexpressing TGFBR1-WT and TGFBR1-S165D on a GPR56 knockdown background. We found that when the 165th amino acid of TGFBR1 is mutated, the TGF-β pathway in HCC cells is inhibited, and their migration and invasion abilities are reduced (Fig. 4I–L). We also used western blot to detect EMT markers and found that reconstitution of TGFBR1 in HCC cells enhances the expression of Vimentin and Occludin while suppressing the expression of E-cadherin, suggesting that GPR56 regulates EMT in HCC cells through TGFBR1 (Supplementary Fig. 4H).

The orthotopic implantation experiments in nude mice demonstrated that downregulation of TGFBR1 expression in the Hep3B-LV-GPR56 group resulted in reduced liver tumor burden, fewer lung metastatic nodules, and extended survival time (Fig. 4M–O). In contrast, overexpression of TGFBR1-WT in the MHCC97H-SH-GPR56 group promoted tumor growth and metastasis, while overexpression of TGFBR1-S165D showed no significant pro-cancer effects (Fig. 4P–R). These results suggest that TGFBR1 is involved in GPR56-mediated HCC metastasis.

### TGF-β1/SMAD3 pathway regulates GPR56 transcription

**Fig. 5:**
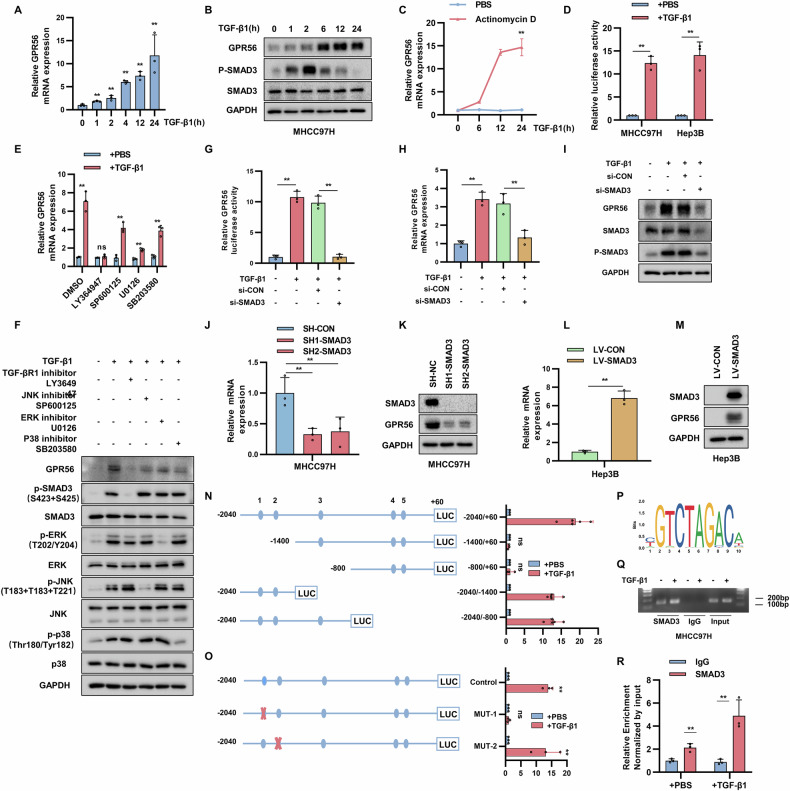
TGF-β1/SMAD3 pathway regulates GPR56 transcription. **A**, **B** Real-time qPCR analysis and Western blot of GPR56 levels of MHCC97H cells stimulated by TGF-β1. **C** mRNA levels of GPR56 in MHCC97H cells treated with TGF-β1 (5 ng/mL) combined with actinomycin D (1 mg/mL). **D** The luciferase activity of MHCC97H and Hep3B cells transfected with pGL4.17-GPR56 (−2040/ + 60) treated with or without TGF-β1. **E**, **F** mRNA levels and protein levels of GPR56 in MHCC97H cells treated with TGF-β1 along with various inhibitors. **G**–**I** Luciferase reporter, real-time qPCR, and Western blot assays were performed to assess GPR56 promoter activity and expression levels. **J**–**M** Real-time qPCR, and Western blot analysis of GPR56 expression. **N**–**O** The luciferase activity in 293 T cells transfected with truncated and mutated GPR56 promoter stimulated with TGF-β1. **P** Schematic of the putative SBE binding motif. **Q**, **R** Chromatin immunoprecipitation assays targeting anti-SMAD3, coupled with real-time qPCR in MHCC97H cells with or without TGF-β1. The representation includes data and error bars, demonstrating the mean ± standard deviation from three separate independent trials. Significance levels are indicated as follows: *p < 0.05, **p < 0.01, ***p < 0.001, and “ns” indicating no statistical significance. The data were analyzed utilizing Student’s t-tests.

Interestingly, our previous observations indicate a time-dependent increase in GPR56 expression within HCC cells following TGF-β1 stimulation (Fig. 3D, E). In order to comprehend the mechanism governing the control of GPR56 expression by TGF-β1, MHCC97H cells underwent treatment with TGF-β1 across different time intervals. Findings indicated that TGF-β1 facilitated augmentation in both the mRNA and protein concentrations of GPR56 in a temporally progressive fashion (Fig. 5A, B). Nevertheless, this enhancement was entirely nullified upon simultaneous exposure to the transcription inhibitor actinomycin D. This suggests that TGF-β1 triggers GPR56 mRNA expression at the transcriptional tier (Fig. 5C). Further confirmation was obtained by investigating the enhancement of GPR56 promoter activity over time under TGF-β1 stimulation in MHCC97H and Hep3B cells (Fig. 5D). In MHCC97H cells, we employed diverse inhibitors targeting the TGF-β signaling pathway, including the SMAD (inhibited by LY364947), ERK (inhibited by U0126), JNK (inhibited by SP600125), and P38 MAPK (inhibited by SB203580) pathways. Our investigation revealed that the elevated expression of GPR56 following TGF-β1 stimulation was entirely abolished by the TGFBR1 inhibitor LY364947. Conversely, inhibitors targeting non-SMAD pathways demonstrated ineffectiveness in altering GPR56 upregulation (Fig. 5E, F). The luciferase reporter gene results showed that only SMAD3 significantly activated the activity of the GPR56 promoter when SMAD2/3/4 were overexpressed separately (Supplementary Fig. 5A). In addition, overexpression of SMAD3 significantly increased GPR56 expression in the presence or absence of TGF-β1 (Supplementary Fig. 5B, C). Conversely, we knocked down SMAD3 by siRNA, thereby eliminating the regulatory effect of TGF-β1 on GPR56 (Fig. 5G–I).

Further confirmation was obtained by separately constructing SMAD3 stable overexpression and knockdown HCC cell lines. Our observations revealed a decrease in the expression levels of GPR56 upon SMAD3 knockdown, while conversely, GPR56 levels increased with SMAD3 overexpression (Fig. 5J–M). Next, we conducted luciferase reporter assays. According to JASPAR analysis, the GPR56 promoter contained 5 predicted SBE domains at positions −1803, −1645, −1156, −387, and −260. Truncated mutants and point mutants of GPR56 promotor suggested that SBE-1 might be accountable for the elevation of GPR56 induced by TGF-β1 stimulation (Fig. 5N, O). Chromatin immunoprecipitation (ChIP) assays confirmed that SMAD3 bound to SBE-1 regions (Fig. 5P–R). Overall, these results suggest that GPR56 is an important target in the TGF-β1 signaling cascade, and the positive feedback loop formed around GPR56 further promotes HCC metastasis.

### Combined treatment of TGFBR1 inhibitor GAL and GPR56-specific small molecule inhibitor DHM dramatically decreased GPR56-driven HCC metastasis

**Fig. 6:**
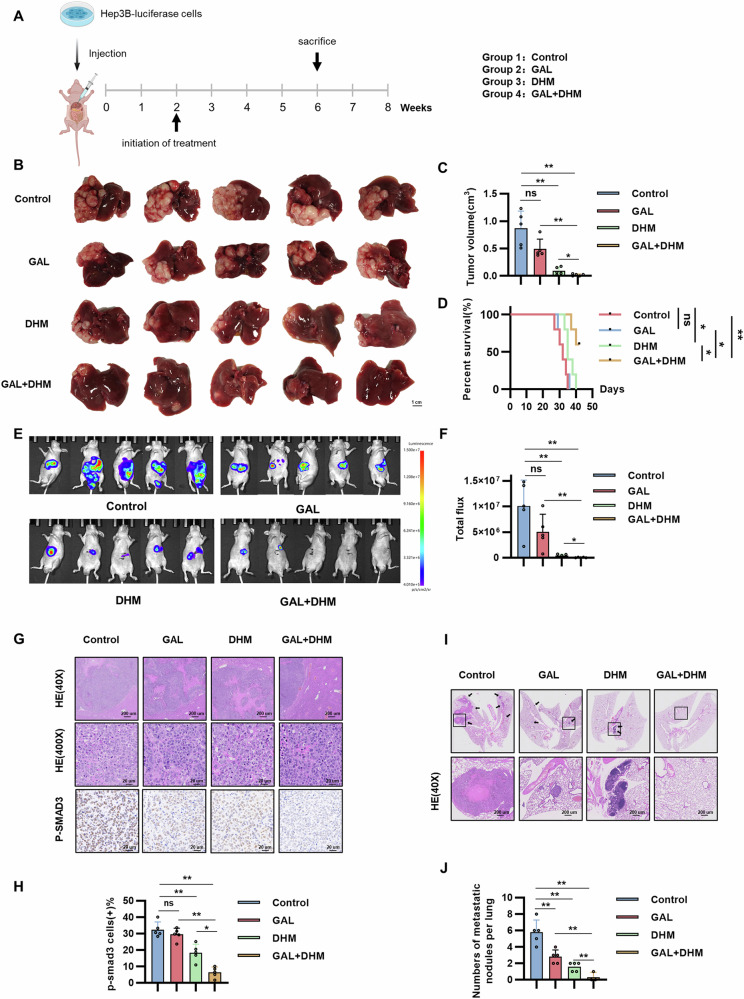
Combined treatment of TGFBR1 inhibitor GAL and GPR56-specific small molecule inhibitor DHM dramatically decreased GPR56-driven HCC metastasis. **A** The diagram of combined treatment in node mice. **B** Representative Gross images. **C** Tumor volume analysis. **D** Survival curves of mice in different treatment groups. **E** Representative Bioluminescence images. **F** Radiance analysis. **G**, **H** Representative HE and p-SMAD3 staining images with result of positive rate. **I**, **J** Representative HE images of lung metastasis and number of lung metastasis nodules. Scale bar: 1 cm (**B**), 200 µm (40×), 20 µm (400×). The representation includes data and error bars, demonstrating the mean ± standard deviation from three separate independent trials. Significance levels are indicated as follows: *p < 0.05, **p < 0.01, ***p < 0.001, and “ns” indicating no statistical significance. The data were analyzed utilizing Student’s t-tests.

The above results demonstrated that TGF-β1 promotes the expression of GPR56 via the canonical TGF-β/SMAD pathway, while GPR56, in turn, facilitates the activation of the TGF-β signaling pathway by binding to and activating TGFBR1. In order to assess whether the combination of the GPR56-specific small molecule inhibitor DHM and the TGFBR1 inhibitor GAL affected GPR56-mediated HCC metastasis, we utilized a mouse model of HCC formed by orthotopic injection of Hep3B-LV-GPR56 cells. After 2 weeks of inoculation with tumor cells, we initiated treatment with DHM, GAL, or their combination for an additional 2 weeks (Fig. 6A). The drugs were previously validated, and our results also showed no apparent toxicity of the combination treatment in mice (Supplementary Fig. 5D). The results showed that treatment with GAL alone was not effective for HCC with high expression of GPR56. Treatment with DHM alone partially reduced the luciferase intensity in the liver, decreased liver tumor burden, and prolonged overall survival compared with controls. Moreover, when DHM was coupled with GAL in a combined regimen, it demonstrated the most potent inhibitory impact (Fig. 6B–F). The levels of p-SMAD3 in tumor tissues were assessed through IHC and WB analysis. Our observations revealed that the intensity of p-SMAD3 staining was lowest in the combination treatment group (Fig. 6G, H and Supplementary Fig. 5E). Furthermore, we assessed the status of lung metastasis and similarly found that the combination treatment with DHM and GAL demonstrated the most potent inhibitory effect on metastatic capability (Fig. 6I. J). These findings suggest that the combination of the DHM and the GAL effectively inhibits GPR56-induced HCC progression.

### GPR56 and SMAD3/p-SMAD3 have similar expression characteristics in HCC

**Fig. 7:**
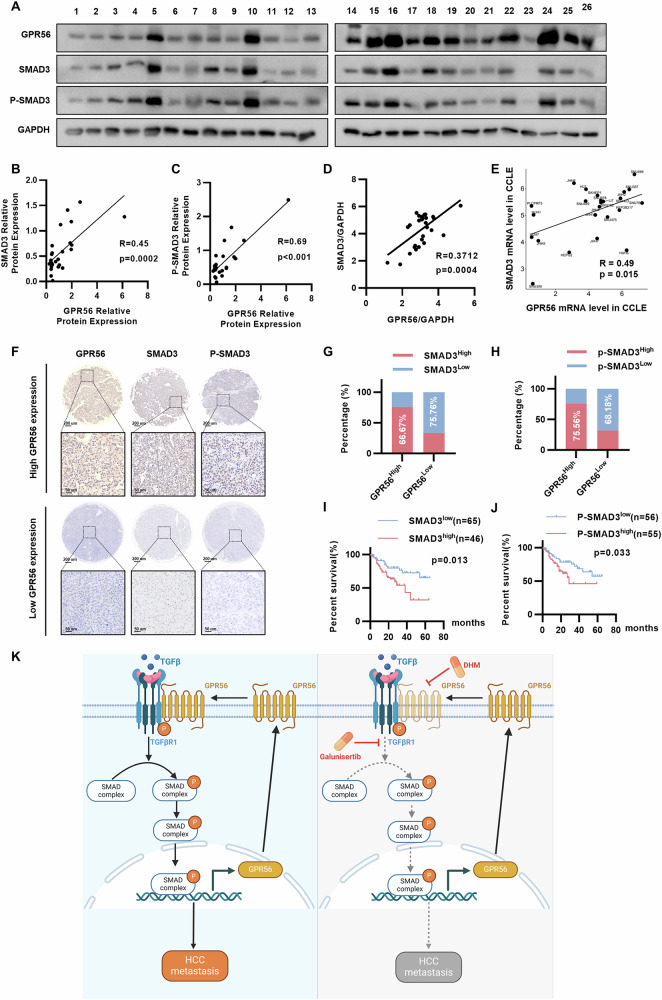
GPR56 and SMAD3/p-SMAD3 have similar expression characteristics in HCC. **A** Western blot of GPR56 and SMAD3/p-SMAD3 in HCC tissues. **B**, **C** Correlation analysis between GPR56 and pSMAD3/SMAD3 protein levels. **D** Correlation analysis between GPR56 and SMAD3 mRNA expression. **E** Correlation analysis between GPR56 and SMAD3 mRNA levels in HCC cell lines sourced from the Cancer Cell Line Encyclopedia database. **F** Representative IHC staining images of GPR56, SMAD3 and p-SMAD3 in Tongji cohort. Scale bar: 200 µm (up), 50 µm (bottom). **G**, **H** Correlation analysis of GPR56 with SMAD3/p-SMAD3 in Tongji cohort. **I**, **J** Kaplan-Meier analysis of SMAD3 or p-SMAD3 expression and overall survival in Tongji cohort. **K** Schematic diagram of positive feedback loop of GPR56 in the TGF-β pathway in HCC. The representation includes data and error bars, demonstrating the mean ± standard deviation from three separate independent trials. Significance levels are indicated as follows: *p < 0.05, **p < 0.01, ***p < 0.001, and “ns” indicating no statistical significance. The data were analyzed utilizing Student’s t-tests.

We evaluated GPR56 and SMAD3/p-SMAD3 protein levels in HCC specimens. The results demonstrated a positive correlation between GPR56 and SMAD3, as well as p-SMAD3 levels in HCC tissues (Fig. 7A–C). And at the mRNA levels, we similarly observed a robust association between GPR56 and SMAD3 (Fig. 7D). Our observation coincides with the outcomes obtained from the Cancer Cell Line Encyclopedia database and is consistent with the results from 11 online datasets, including TCGA-LIHC (Fig. 7E and Supplementary Fig. 6A). To further corroborate this correlation, we investigated the correlation between GPR56 and SMAD3 and pSMAD3 in Tongji cohort of HCC patients. Representative IHC staining images of GPR56, SMAD3, and p-SMAD3 are illustrated (Fig. 7F). The analysis showed that in Tongji cohort, the expression level of GPR56 showed a significant positive correlation with the expression levels of SMAD3 and p-SMAD3 (Fig. 7G, H). Moreover, high expression levels of both proteins indicated poor prognosis (Fig. 7I, J). Overall, our findings confirm the positive correlations between GPR56, SMAD3, and p-SMAD3 in HCC patients.

## Discussion

Genomic and proteomic studies have indicated that GPR56 is overexpressed and plays a pro-carcinogenic role in various cancers, including breast cancer, epithelial ovarian cancer, esophageal cancer, and colorectal cancer [17, 19, 21, 29]. On the contrary, certain studies have indicated that GPR56 expression is downregulated in metastatic melanoma and glioma, thereby acting as a tumor suppressor [26, 30]. Thus, the role of GPR56 in cancer may exhibit tissue specificity. With its large N-terminal fragment (NTF), GPR56 participates in a multitude of cell-cell and cell-extracellular matrix interactions, thus playing a key role in complex signaling pathways in cancer [31]. Our investigation disclosed that the levels of GPR56 expression in cancerous tissues of HCC patients were markedly elevated compared to those in neighboring non-cancerous tissues, and it correlated with differentiation degree, tumor number, macrovascular invasion, and BCLC stage. Moreover, experiments conducted in vitro and in vivo conclusively illustrated that GPR56 promotes the ability of HCC cells to invasion and metastasis, and its pro-metastatic effect is attenuated when GPR56 is inhibited. These results strongly indicate the critical involvement of GPR56 in HCC metastasis.

In our earlier investigation, we observed a notable downregulation of GPR56 in LM3 cells that knockdown SMAD3 [8]. In this study, we further confirmed that GPR56 is directly transcribed by p-SMAD3 activated by TGF-β1. Additionally, through RNA-seq and proteomic analysis, we discovered that GPR56 can to bind and activate TGFBR1, thereby feedback activating the TGF-β signaling pathway.

Numerous studies have validated the association between activation of the TGF-β signaling pathway and unfavorable prognosis in cancer [6, 32, 33]. Upon activation of TGF-βRII and TGF-βRI, phosphorylation of SMAD2/3 ensues, facilitating their interaction with SMAD4. This complex formation then modulates the transcriptional activity of target genes, either activating or inhibiting their expression [34]. Once tumor cells evade the inhibitory effects of TGFβ signaling through genetic or epigenetic changes, overexpression of TGFβ can promote tumor initiation, progression, and even metastasis [35]. The significance of the TGF-β signaling pathway in HCC progression is underscored by these investigations. Currently, targeting the TGF-β signaling pathway for cancer treatment has become a promising strategy [6, 36]. Several small molecule inhibitors targeting TGF-β receptors I/II have been developed, with three currently undergoing clinical development [36–38]. Galunisertib (LY2157299, GAL) stands out as a selective ATP-competitive inhibitor of TGFBR1(ALK5) and is the only inhibitor of the TGF-β pathway used in clinical studies in HCC patients (NCT01246986) [39, 40]. While GAL has demonstrated efficacy in inhibiting tumor progression in preclinical models of pancreatic cancer and HCC, the use of TGF-β inhibitors can lead to a range of side effects, including immunosuppression, hepatotoxicity, and cardiotoxicity, which may limit the use of GAL [41].

In our study, we observed that GAL did not exhibit significant efficacy against HCC with high GPR56 expression. Considering the mechanism of action of GAL, we speculated that this might be associated with the activation of TGFBR1 induced by GPR56, suggesting a potential competitive interaction between GPR56 and GAL. Therefore, we used the small molecule inhibitor DHM to selectively target GPR56 [42]. In our animal experiments, the combination of DHM and GAL indeed showed superior efficacy compared to monotherapy in HCC with high GPR56 expression, significantly suppressing HCC metastasis. These findings highlight a novel strategy targeting GPR56-induced HCC metastasis.

## Supplementary information

Supplementary information

uncropped Western Blots

## Data Availability

The data that support the findings of this study are available from the corresponding authors upon reason-able request. The raw sequence data have been deposited in Genome Sequence Archive in National Genomics Data Center, Beijing Institute of Genomics (https://ngdc.cncb.ac.cn/gsa-human) with Project Accession No. PRJCA029974.
