# Supplementary material for: GPR56 facilitates hepatocellular carcinoma metastasis by promoting the TGF-β signaling pathway
Source: Cell Death Dis. 2024 Oct 1;15(10):715. doi: 10.1038/s41419-024-07095-6 (PMC11445230; doi:10.1038/s41419-024-07095-6)
Supplement: Supplementary file 1 — Supplementary information [file 41419_2024_7095_MOESM1_ESM.docx]

**GPR56 orchestrates hepatocellular carcinoma metastasis by promoting TGF-β signaling pathway**

**Supplementary Figure 1**


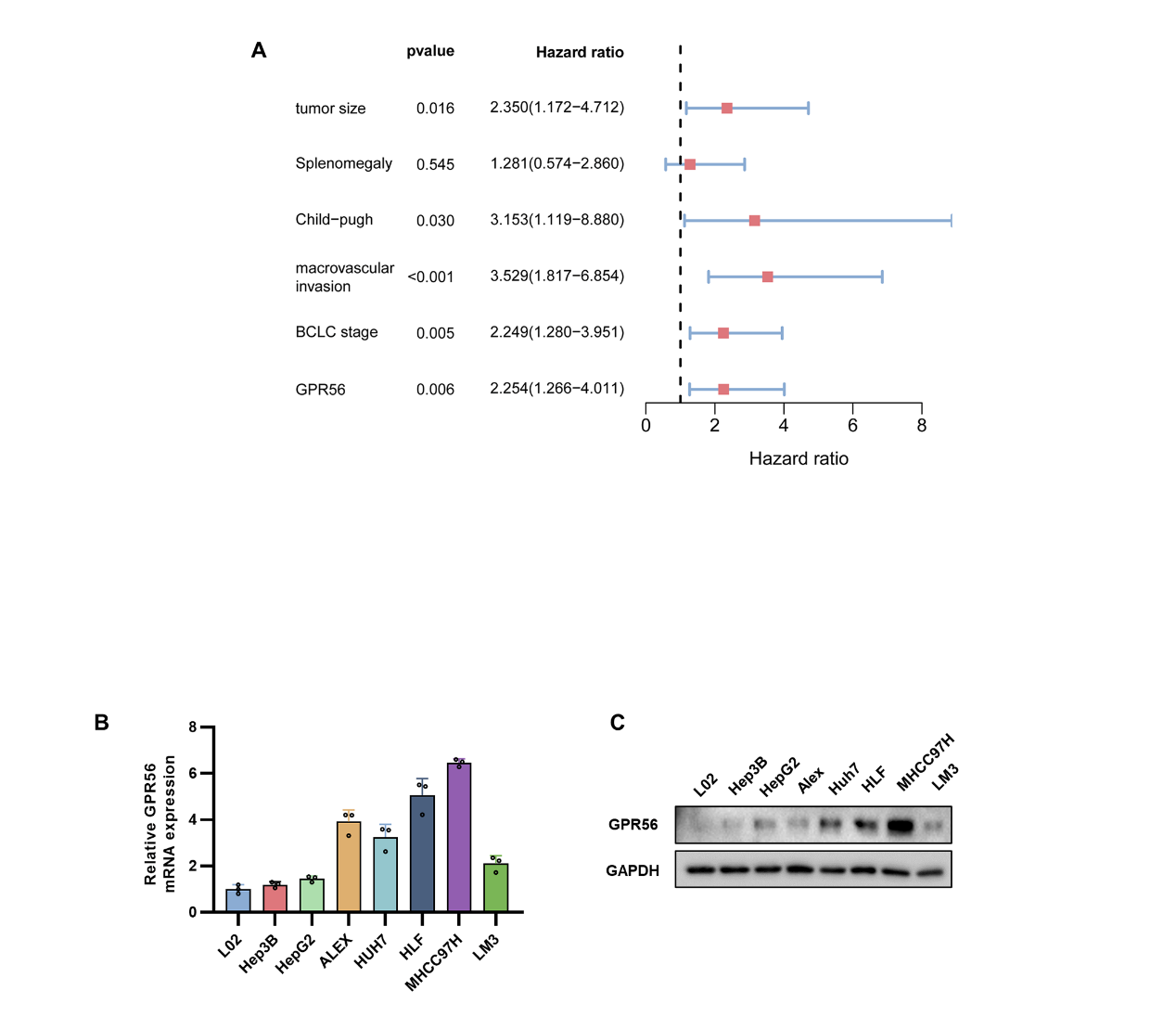


**Supplementary Figure 1, Increased GPR56 expression demonstrated a correlation with clinical outcomes among HCC patients.** (A) Univariate regression analyses from the Tongji cohort in the forest plot. (B-C) The mRNA and protein of GPR56 expression levels in HCC cell lines and normal liver tissue.

**Supplementary Figure 2**


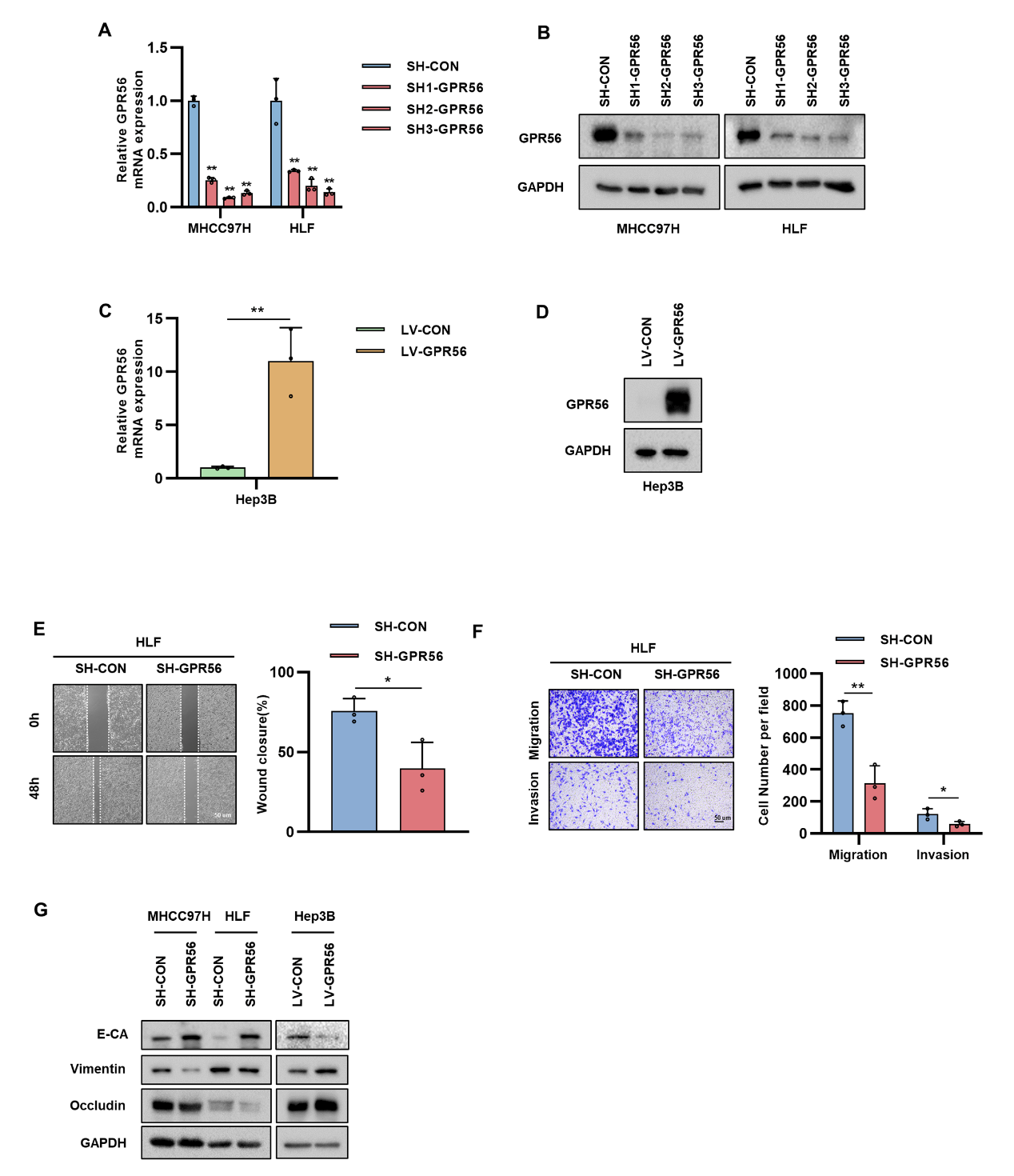


**Supplementary Figure 2, GPR56 facilitated the metastatic progression of HCC in vitro.** (A-B) Real-time qPCR and WB assessed the effectiveness of GPR56 knockdown in MHCC97H and HLF cells. (C-D) Real-time qPCR assessed the effectiveness of GPR56 overexpression in Hep3B cells. (E) Representative images and the rate of wound closure from HLF SH-GPR56 cells. (F) Representative images and the count of cells in HLF SH-GPR56 cells. (G) WB analysis of EMT markers in MHCC97H and HLF with GPR56 knockdown and Hep3B with GPR56 overexpression.

**Supplementary Figure 3**


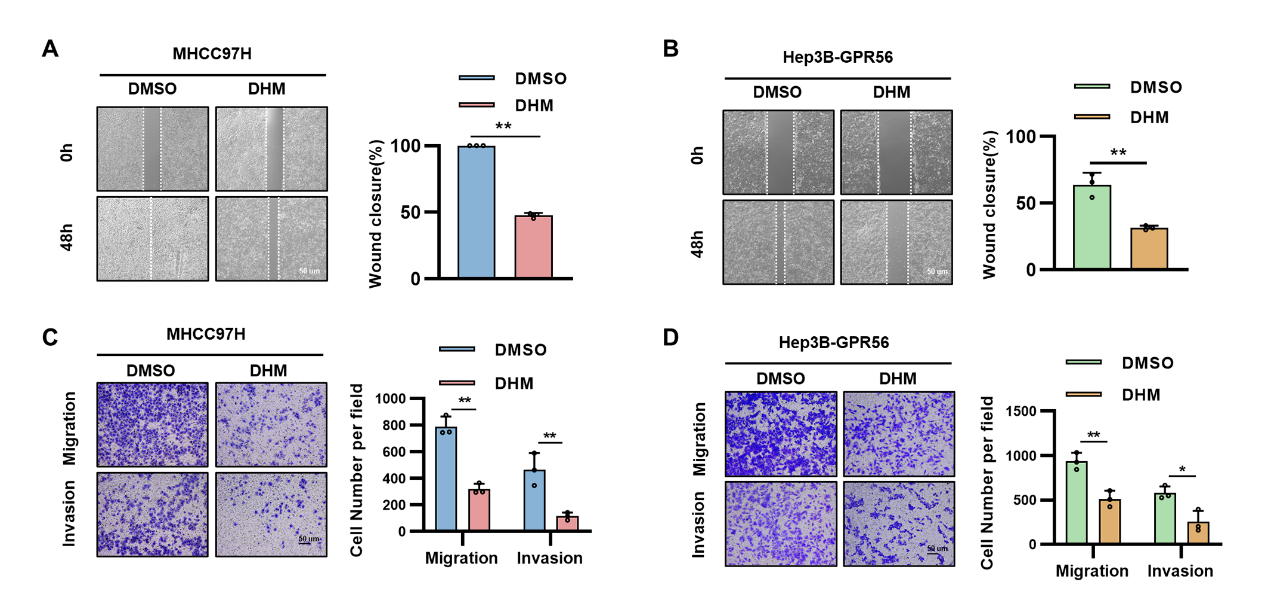


**Supplementary Figure 3,** **DHM inhibits HCC invasion and metastasis in vitro.**

(A-B) Representative images and the rate of wound closure in DHM treatment cells. (C-D) Representative images and the count of cells in DHM treatment cells.

**Supplementary Figure 4**


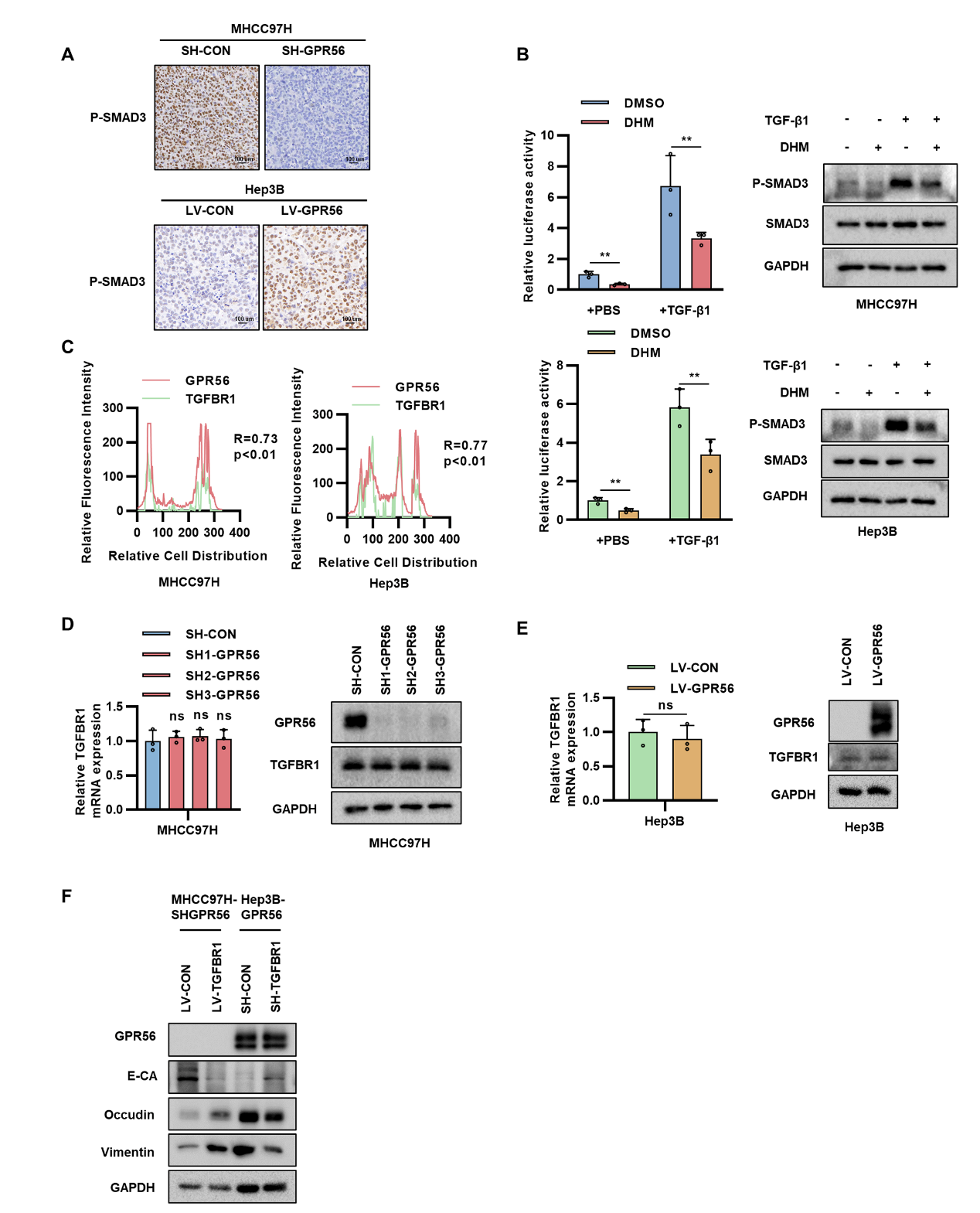


**Supplementary Figure 4, GPR56 exhibited upregulation of the TGF-β pathway and interaction with TGFBR1.** (A) Representative IHC staining of p-SMAD3 in the mouse livers. (B) SBE luciferase activity in MHCC97H and Hep3B stimulated with TGF-β or DHM. (C) Fluorescence intensities in the nucleus in MHCC97H and Hep3B. (D-E) The mRNA and protein levels of TGFBR1 in MHCC97H SH-GPR56 and Hep3B LV-GPR56 cells. (F) WB analysis of EMT markers in MHCC97H-SHGPR56 with TGFBR1 overexpression and Hep3B-GPR56 with TGFBR1 knockdown.

**Supplementary Figure 5**


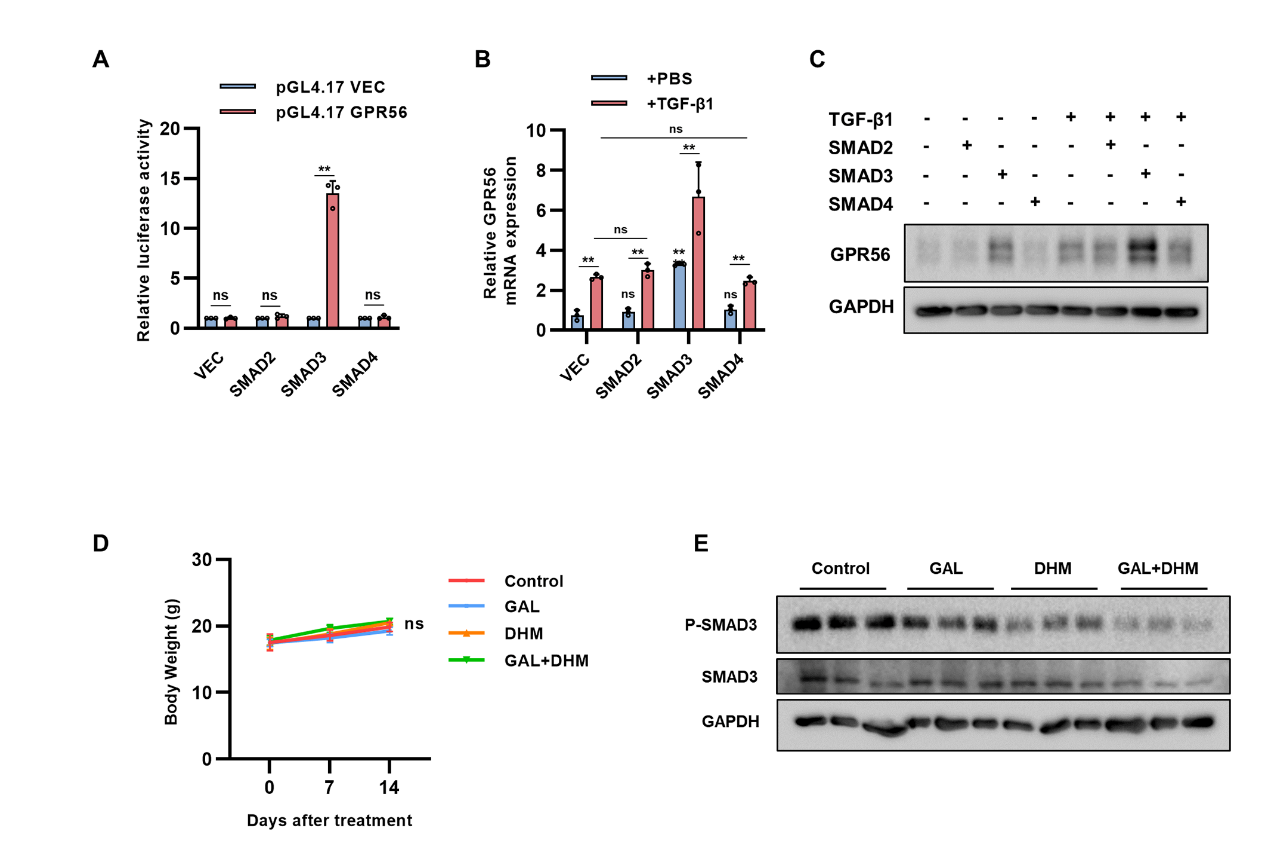


**Supplementary Figure 5, TGF-β1/SMAD3 pathway regulates GPR56 transcription.** (A) Luciferase activity in MHCC97H cells transfected with pGL4.17-GPR56 and SMAD2/3/4. (B-C) Real-time qPCR analysis and WB were conducted to assess the expression levels of GPR56 in MHCC97H cells transfected with SMAD2/3/4 with or without TGF-β1. (D) Body weight of node mice in different drug treated groups. (E) Western blotting analysis of the expression of p-SMAD3, SMAD3 and GAPDH in BALB/c nude mice xenograft.

**Supplementary Figure 6**


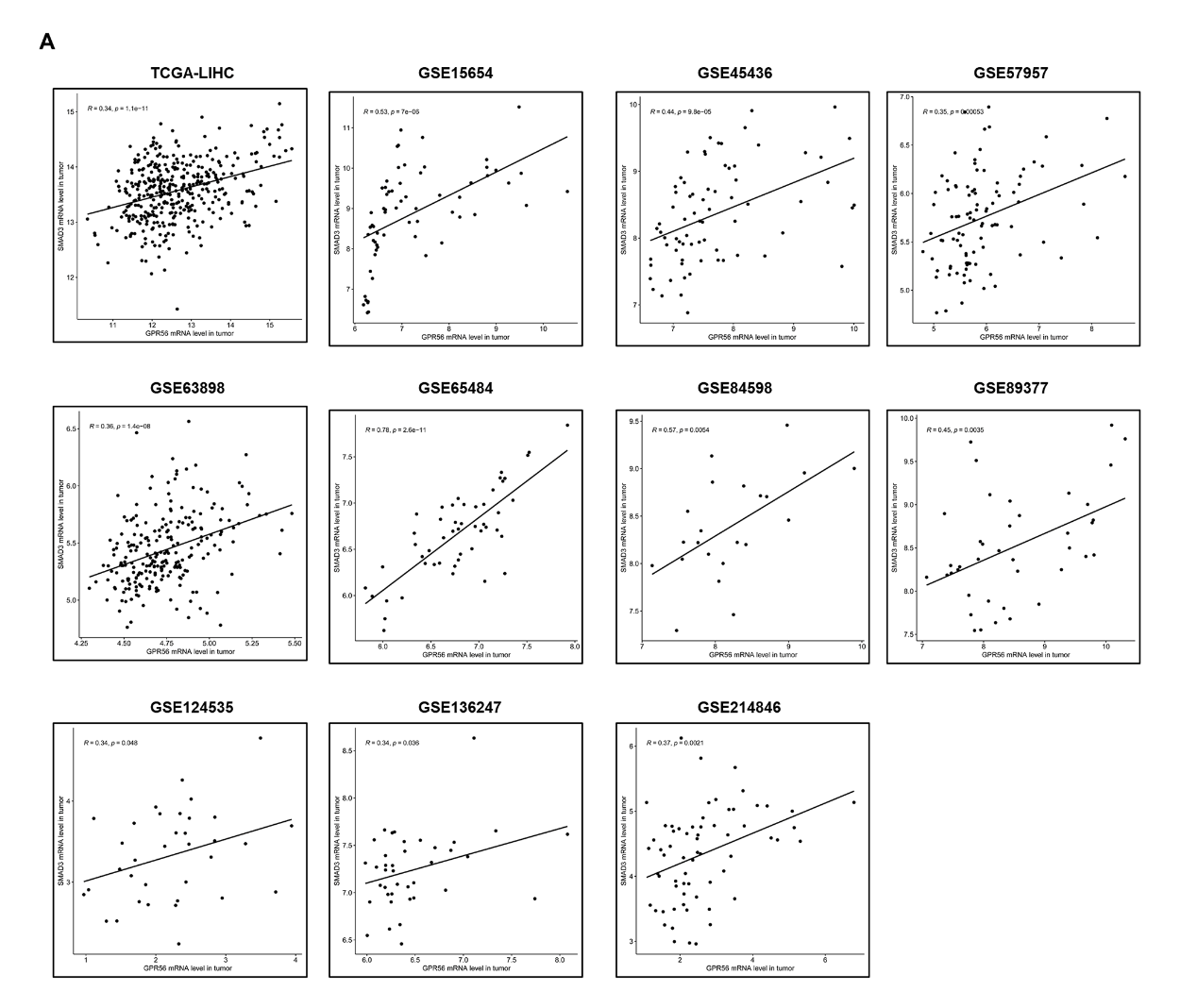


**Supplementary Figure 6,** **GPR56 and SMAD3/p-SMAD3 have similar expression characteristics in HCC. (**A) The correlation between GPR56 expression and SMAD3 expression in liver cancer in 11 online datasets.

**Supplementary Table S1. Correlation between clinicopathological features and GPR56 expression in 123 HCC cases**

| **Clinicopathological variables** | **Tumor GPR56 expression** | | ***P***  ***Value*** |
| --- | --- | --- | --- |
|  | **Low expression**  **(n=72)** | **High expression**  **(n=51)** |  |
| ***Differentiation**** |  |  |  |
| High grade | 61 | 20 |  |
| Low grade | 11 | 31 | **<0.0001** |
| ***Gender*** |  |  |  |
| Male | 57 | 46 |  |
| Female | 15 | 5 | 0.1377 |
| ***Age*** |  |  |  |
| <60 years | 61 | 36 |  |
| >60 years | 11 | 15 | 0.0739 |
| ***AFP*** |  |  |  |
| <400 | 37 | 29 |  |
| >400 | 35 | 22 | 0.5856 |
| ***Tumor size*** |  |  |  |
| <5 cm | 28 | 15 |  |
| >5 cm | 44 | 36 | 0.3386 |
| ***Child-pugh*** |  |  |  |
| A+B | 71 | 46 |  |
| C | 1 | 4 | 0.1577 |
| ***Tumor number**** |  |  |  |
| Single | 59 | 33 |  |
| Multiple | 13 | 18 | **0.0363** |
| ***Cirrhosis*** |  |  |  |
| Absent | 23 | 12 |  |
| Present | 49 | 39 | 0.4175 |
| ***Tumor encapsulation*** |  |  |  |
| Absent | 68 | 46 |  |
| Present | 4 | 5 | 0.4871 |
| ***TNM stage**** |  |  |  |
| I-II | 51 | 39 |  |
| III | 21 | 12 | 0.5401 |
| ***Macrovascular Invasion*** |  |  |  |
| Absent | 67 | 40 |  |
| Present | 5 | 11 | **0.0276** |
| ***Microvascular Invasion**** |  |  |  |
| Absent | 66 | 43 |  |
| Present | 6 | 8 | 0.2538 |
| ***BCLC stage*** |  |  |  |
| A | 52 | 26 |  |
| B+C | 20 | 25 | **0.0224** |
| ***Tumor capsule*** |  |  |  |
| Absent | 32 | 25 |  |
| Present | 40 | 27 | 0.718 |

**Supplementary Table S2. Reagents used in cells in this study.**

| Reagents | Source |
| --- | --- |
| TGF-β1 | Novoprotein, Shanghai, China (5ng/mL) |
| TGFBR1 inhibitor LY364947 | MedChemExpress (10μM) |
| ERK inhibitor U0126 | MedChemExpress (10μM) |
| JNK inhibitor SP600125 | MedChemExpress (20μM) |
| p38 inhibitor SB203580 | MedChemExpress (20μM) |
| MEK inhibitor PD098059 | MedChemExpress (10μM) |
| Galunisertib | Selleck (10μM) |
| Dihydromunduletone | MedChemExpress (20μM) |

| Antigen | Manufacturer | Catalog Number | Application |
| --- | --- | --- | --- |
| GPR56 | Affinity | DF2753 | 1:100 for IHC |
| GPR56 | Abcam | ab302909 | 1:1000 for WB |
| GAPDH | MedChemExpress | HY-P80137 | 1:10000 for WB |
| TGFBR1 | Abcam | ab235578 | 1:1000 for WB |
| p-TGFBR1 | Abcam | ab112095 | 1:1000 for WB |
| SMAD3 | CST | C67H9 | 1:1000 for WB |
| p-SMAD3 | HUABIO | ET1609-41 | 1:1000 for WB |
| SMAD2 | CST | D43B4 | 1:1000 for WB |
| p-SMAD2 | HUABIO | SD207-1 | 1:1000 for WB |
| FLAG | MedChemExpress | HY-P80111 | 1:1000 for IP |
| HA | MedChemExpress | HY-P80948 | 1:1000 for IP |
| GST-tag | MedChemExpress | HY-P80148 | 1:1000 for WB |
| E-CAD | Proteintech | 20874-1-AP | 1:1000 for WB |
| Pan Phospho | Abclone | AP0893 | 1:1000 for WB |
| Vimentin | (Bei jing Solarbio Science & Technology Co., Ltd.)，Solarbio | K002388P | 1:1000 for WB |
| Occludin | Proteintech | 27260-1-AP | 1:1000 for WB |
| Goat Anti-Mouse IgG-Fc Secondary Antibody (HRP) | Sino Biological Inc. (Beijing, China) | SSA007 | 1:5000 for WB |
| Goat Anti-Rabbit IgG-Fc Secondary Antibody (HRP) | Sino Biological Inc. (Beijing, China) | SSA003 | 1:5000 for WB |

**Supplementary Table S3. Primary antibodies and secondary antibodies used in this study.**

**Supplementary Table S4. Primer sequences and SHRNAs used in this study.**

| **Primers for RT-qPCR** | | |
| --- | --- | --- |
| Gene | Forward primer (5'-3') | Reverse primer (5'-3') |
| GAPDH | GATGGGTGTGAACCACGAGAA | GGGCCATCCACAGTCTTCTG |
| GPR56 | CCAGCGGAACCAGACACAC | TCTTCGGAGTTCTCGATGGAG |
| TGFBR1 | ACGGCGTTACAGTGTTTCTG | GCACATACAAACGGCCTATCTC |
| SMAD3 | TGGACGCAGGTTCTCCAAAC | CCGGCTCGCAGTAGGTAAC |
| **Primers for SHRNA** | | |
| SH-VEC | TTCTCCGAACGTGTCACGT | |
| SH1-GPR56 | GCGTTCAATCTTGACCTTGAA | |
| SH2-GPR56 | CCATCATCTTGGCTGTGCATA | |
| SH3-GPR56 | GACTTCTTGCTGAGTGACAAA | |
| SH1-SMAD3 | GAGCCTGGTCAAGAAACTCAA | |
| SH2-SMAD3 | TGAGCAGAACAGGTAGTATTA | |
| si-SMAD3 | GAGCCTGGTCAAGAAACTCAA | |
| **Primers for Chip** | | |
| Gene | Forward primer (5'-3') | Reverse primer (5'-3') |
| GPR56 | TCACTGGAGCTGGGCAGT | TGGGCCAAGAGTCACAAACT |
