# Supplementary material for: GPR56 facilitates hepatocellular carcinoma metastasis by promoting the TGF-β signaling pathway
Source: Cell Death Dis. 2024 Oct 1;15(10):715. doi: 10.1038/s41419-024-07095-6 (PMC11445230; doi:10.1038/s41419-024-07095-6)

Raw images of the immunoblotting experiments

FIGURE 1D

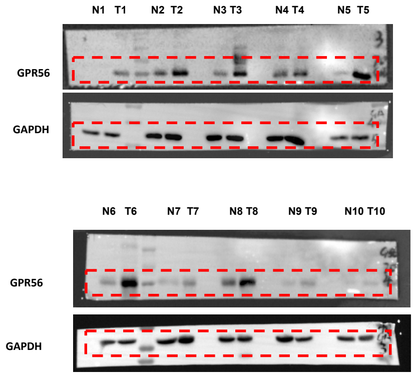

FIGURE 3D

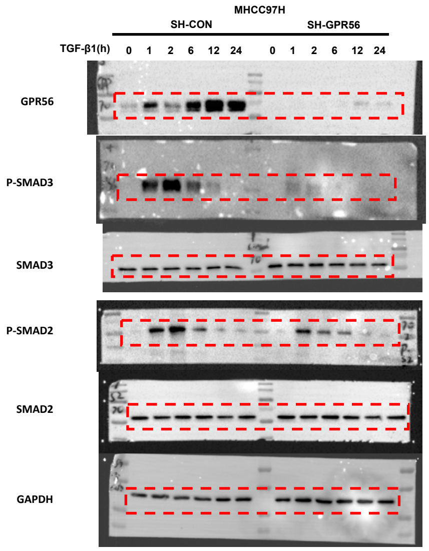

FIGURE 3E

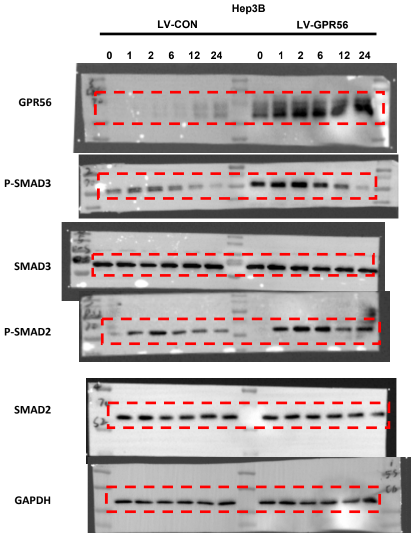

FIGURE 3H

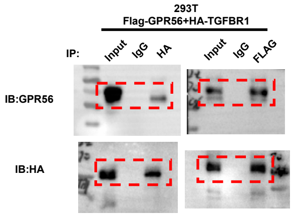

FIGURE 3I

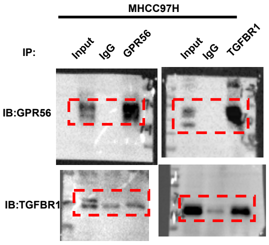

FIGURE 3J

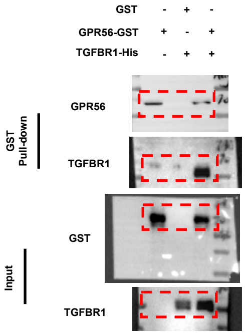

FIGURE 3L

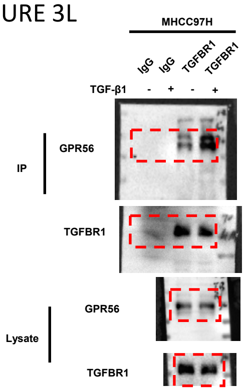

FIGURE 3M

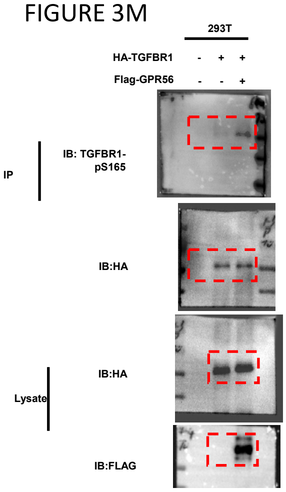

FIGURE 4A

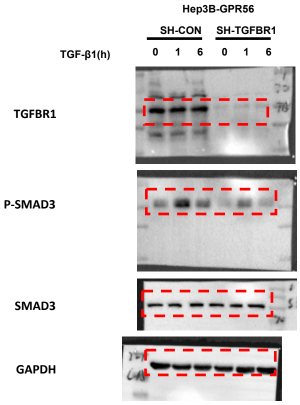

FIGURE 4E

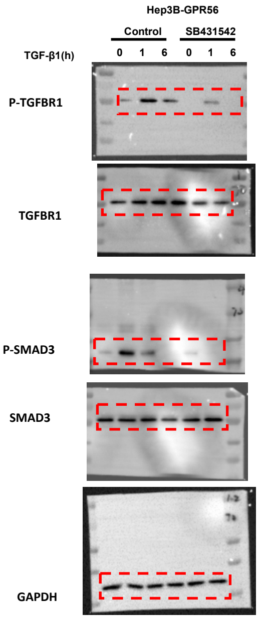

FIGURE 4I

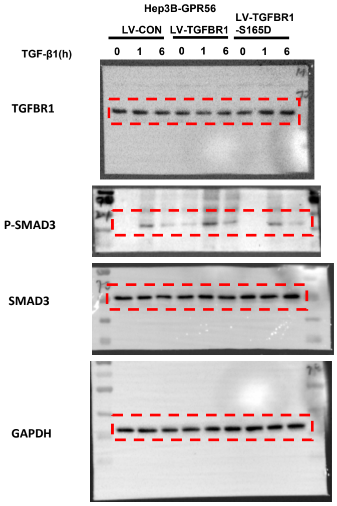

FIGURE 5B

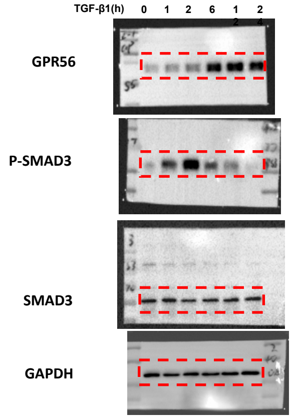

FIGURE 5F

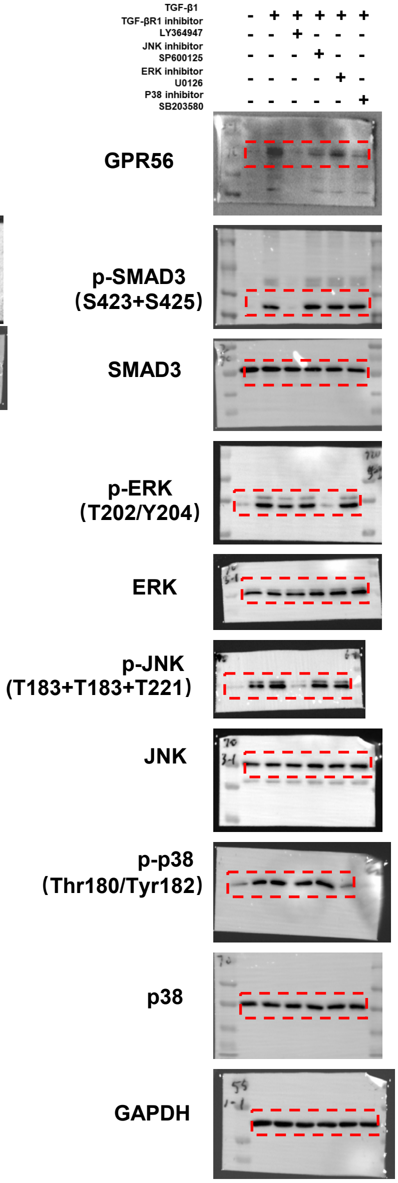

FIGURE 5I

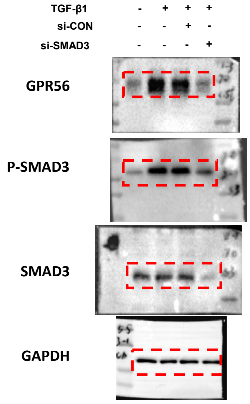

FIGURE 5K

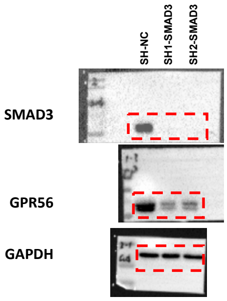

FIGURE 5M

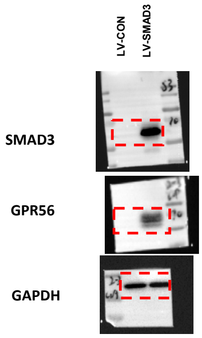

FIGURE 7A

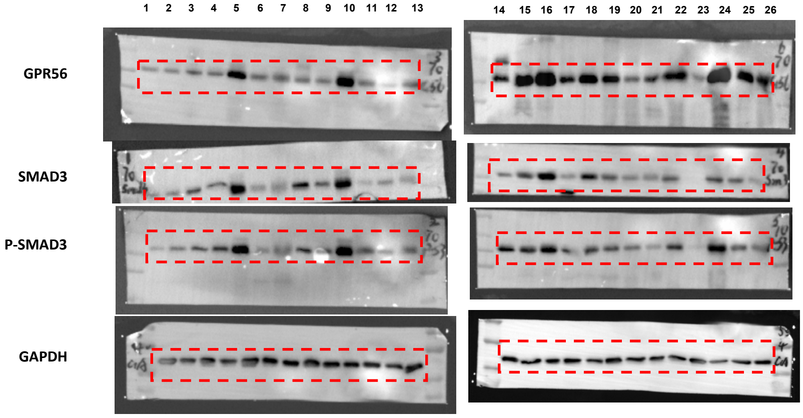

SUPPLEMENTRAY FIGURE 1C

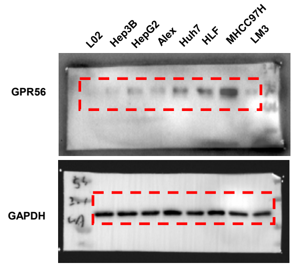

SUPPLEMENTRAY FIGURE 2B

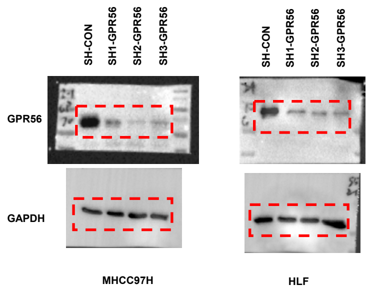

SUPPLEMENTRAY FIGURE 2D

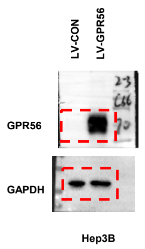

SUPPLEMENTRAY FIGURE 2G

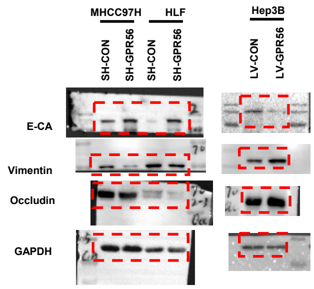

SUPPLEMENTRAY FIGURE 4B

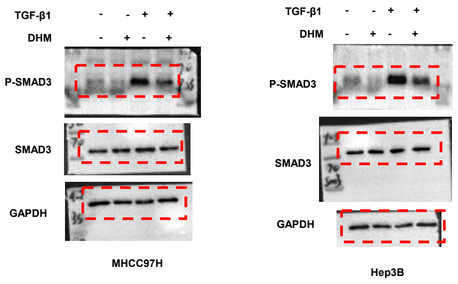

SUPPLEMENTRAY FIGURE 4D

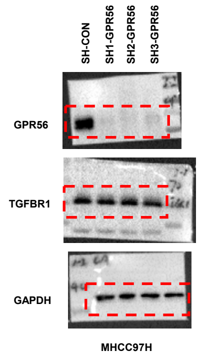

SUPPLEMENTRAY FIGURE 4E

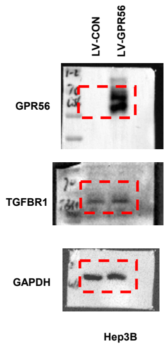

SUPPLEMENTRAY FIGURE 4F

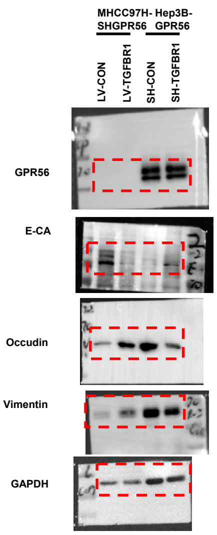

SUPPLEMENTRAY FIGURE 5C

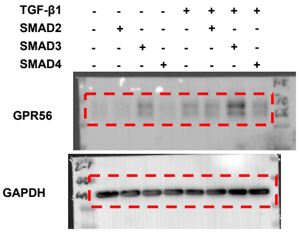

SUPPLEMENTRAY FIGURE 5E

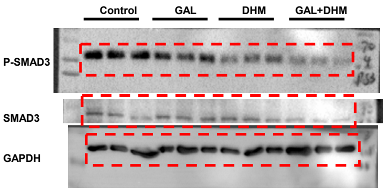

Supplement: Supplementary file 2 — uncropped Western Blots [file 41419_2024_7095_MOESM2_ESM.pdf]
